# Noninvasive ventilation immediately after extubation improves weaning outcome after acute respiratory failure: a randomized controlled trial

**DOI:** 10.1186/cc12549

**Published:** 2013-03-04

**Authors:** Susana R Ornico, Suzana M Lobo, Helder S Sanches, Maristela Deberaldini, Luciane T Tófoli, Ana M Vidal, Guilherme P Schettino, Marcelo B Amato, Carlos R Carvalho, Carmen S Barbas

**Affiliations:** 1 Department of Critical Care, Hospital de Base de São José do Rio Preto, Av.Brigadeiro Faria Lima 5544, Sao Jose do Rio Preto, Post Code: 150921-240, Brazil; 2 Department of Pulmonary and Critical Care, University of Sao Paulo Medical School, Av. Dr Eneas de Carvalho Aguiar 44-2 andar, Sao Paulo, Post Code: 05403-900, Brazil

## Abstract

**Introduction:**

Noninvasive ventilation (NIV), as a weaning-facilitating strategy in predominantly chronic obstructive pulmonary disease (COPD) mechanically ventilated patients, is associated with reduced ventilator-associated pneumonia, total duration of mechanical ventilation, length of intensive care unit (ICU) and hospital stay, and mortality. However, this benefit after planned extubation in patients with acute respiratory failure of various etiologies remains to be elucidated. The aim of this study was to determine the efficacy of NIV applied immediately after planned extubation in contrast to oxygen mask (OM) in patients with acute respiratory failure (ARF).

**Methods:**

A randomized, prospective, controlled, unblinded clinical study in a single center of a 24-bed adult general ICU in a university hospital was carried out in a 12-month period. Included patients met extubation criteria with at least 72 hours of mechanical ventilation due to acute respiratory failure, after following the ICU weaning protocol. Patients were randomized immediately before elective extubation, being randomly allocated to one of the study groups: NIV or OM. We compared both groups regarding gas exchange 15 minutes, 2 hours, and 24 hours after extubation, reintubation rate after 48 hours, duration of mechanical ventilation, ICU length of stay, and hospital mortality.

**Results:**

Forty patients were randomized to receive NIV (20 patients) or OM (20 patients) after the following extubation criteria were met: pressure support (PSV) of 7 cm H_2_O, positive end-expiratory pressure (PEEP) of 5 cm H_2_O, oxygen inspiratory fraction (FiO_2_) ≤ 40%, arterial oxygen saturation (SaO_2_) ≥ 90%, and ratio of respiratory rate and tidal volume in liters (f/TV) < 105. Comparing the 20 patients (NIV) with the 18 patients (OM) that finished the study 48 hours after extubation, the rate of reintubation in NIV group was 5% and 39% in OM group (*P *= 0.016). Relative risk for reintubation was 0.13 (CI = 0.017 to 0.946). Absolute risk reduction for reintubation showed a decrease of 33.9%, and analysis of the number needed to treat was three. No difference was found in the length of ICU stay (*P *= 0.681). Hospital mortality was zero in NIV group and 22.2% in OM group (*P *= 0.041).

**Conclusions:**

In this study population, NIV prevented 48 hours reintubation if applied immediately after elective extubation in patients with more than 3 days of ARF when compared with the OM group.

**Trial Registration number:**

ISRCTN: 41524441.

## Introduction

In critically ill adult patients, particularly patients with chronic obstructive pulmonary disease (COPD), early noninvasive ventilation weaning is associated with the decrease of mortality, ventilator-associated pneumonia, length of stay in the intensive care unit and hospital, total duration of mechanical ventilation, and duration of invasive ventilation [1-6]. The effect of this benefit is not so clear in ICU mixed populations [1-6].

The trials that analyzed the role of noninvasive ventilation (NIV) in weaning outcome used different weaning protocols, distinct moments of discontinuation of mechanical ventilation, as well as different methods of use of noninvasive ventilation [1-6].

Some authors, such as Nava and colleagues [7], used early NIV as soon as COPD patients rested their respiratory muscles, although they were not ready yet to tolerate a spontaneous breathing trial. Others, however, used NIV after COPD patients met extubation criteria [8]. In some studies, NIV support was delivered continuously [7,9-11], but intermittently in others [12]. The interface used to apply NIV varied from face [13] to nasal mask [14]. The level of NIV support also differed, as well as its mode of application from pressure modes [13,15-17] to proportional assisted modes [14]. The optimal timing for transitioning patients to NIV, as well as its use in a mixed ICU population, remains to be determined [1-6]. We hypothesized that early application of NIV with supplemental oxygen immediately after elective or planned extubation in patients with more than 3 days of acute respiratory failure of various etiologies requiring mechanical ventilation would decrease the need for reintubation compared with the oxygen mask.

The objective of this randomized prospective trial was to compare the efficacy of NIV with the oxygen mask in preventing reintubation if NIV was used immediately after elective extubation in patients with acute respiratory failure requiring mechanical ventilation for more than 72 hours. The secondary objectives were to evaluate the differences between the study groups concerning ICU length of stay and hospital mortality.

## Materials and methods

### Patients' selection, management, and randomization

The study was carried out over a 12-month period in a single center of a 24-bed adult general ICU in a University Hospital. The study was approved by the Ethics Committee of the University of São Paulo and was registered under ISRCTN number 41524441. It was performed in accordance with the ethical standards laid down in the 1964 Declaration of Helsinki. Informed consent was obtained before extubation from the closest next-of-kin for each patient. Inclusion criteria: patients with acute respiratory failure (PaO_2_/FiO_2 _ratio ≤ 300 or PaCO_2 _≥ 50 mm Hg at intubation); invasive mechanical ventilation for a period longer than 72 hours administered by orotracheal tube; weaning from invasive mechanical ventilation by using the ICU weaning protocol; absence of contraindications for the use of NIV, which were defined as: cardiac or respiratory arrest, severe encephalopathy (Glasgow coma scale < 10), bleeding of the upper gastrointestinal tract, hemodynamic instability or severe arrhythmia, facial surgery or trauma or deformity, severe upper-airway obstruction, inability to cooperate or protect the airways, inability to cough or clear respiratory secretions, absence of a gag reflex, and severe gastric distention. Exclusion criteria: younger than 18 years, pregnancy, and patient's refusal to participate in the study. We considered COPD patients those with any known history of underlying COPD, regardless of the etiology of respiratory failure.

### Weaning protocol

After 72 hours of invasive mechanical ventilation, patients were evaluated on a daily basis, and weaning was considered when the criteria listed were met: cardiovascular stability (hemoglobin ≥ 8 g/dl, no severe arrhythmia), hemodynamic stability (absence of vasopressors or vasopressors in doses ≤ 5 μg/kg/min), gas-exchange stability (PaO_2 _≥ 60 mm Hg with SaO_2 _≥ 90% and FiO_2 _≤ 40%), pulmonary mechanics stability (control of pulmonary edema, atelectases, secretions, and bronchospasm), neurologic stability (GCS, > 10), electrolytic stability (control of alkalosis, acidosis, calcium, magnesium, phosphorus, sodium, potassium), preserved cough reflex, rapid and shallow breathing rate (f/TV, ratio of respiratory rate and tidal volume in liters) < 105, absence of respiratory infection (defined as a new or progressive pulmonary infiltrate at chest radiograph, temperature < 36°C or > 37.8°C, leukocytosis (leukocytes > 12,000/ml) or leukopenia (leukocytes < 4,000/ml)).

The weaning protocol was based on a gradual reduction of pressure-support ventilation mode (PSV) combined with one assist/control breath per minute of synchronized intermittent mandatory ventilation (SIMV). The adjustments of the mechanical ventilator were as follows: PSV to obtain an expiratory tidal volume of 8 ml/kg; SIMV with a respiratory rate of 1 and a tidal volume of 8 ml/kg, FiO_2 _≤ 40%, PEEP required to obtain SaO_2 _≥ 90%, and pressure sensitivity of 0.5 cm H_2_O. The pressure-support level was decreased by 2 cm H_2_O every 2 hours until a PSV of 7 cm H_2_O was reached. If f/TV > 105, PSV was increased to the previous value for a minimum period of 6 hours, after which the protocol was then resumed. In the cases in which PEEP exceeded 5 cm H_2_O, it was gradually decreased by 2 cm H_2_O every 6 hours until a value of 5 cm H_2_O was reached. The patient was considered ready for extubation, which was carried out in PSV of 7 cm H_2_O, PEEP 5 cm H_2_O, SaO_2 _≥ 90%, FiO_2 _< 40%, and f/TV < 105.

### Randomization

*A priori*, we prepared blocks of 40 slips of paper, each of which identified by a number (from 1 to 20) and the assigned study group (NIV or OM) written on them. These slips were folded and put in an opaque envelope. Patients were randomized immediately before elective extubation: one of the authors in the presence of other members of ICU staff drew one of the folded slips from the opaque envelope to determine the designated study group of that patient. After that, the researchers triggered the next steps of the protocol.

### Noninvasive ventilation group

After randomization and still under invasive mechanical ventilation, patients assigned to the NIV group were informed of the procedure they were about to undergo. After extubation, a respiratory physiotherapist adjusted a silicone nasal mask (Respironics) to the patient's face, holding it for a few minutes and then fixing it firmly and comfortably to the patient's face. The size of the mask was tailored to each patient, and local treatment was used in case of pain or erythema caused by skin compression of the mask. NIV was administered with a BiPAP (Ventilatory Support System; Respironics Inc, Murrysville, PA, USA) device model S/T-D 30 or S/T-D, in spontaneous mode for a continuous period of 24 hours. After this period, the nasal mask was replaced by a nebulization oxygen mask with a flow of 5 L/min. For all patients, the initial EPAP and delta IPAP values were 8 cm H_2_O and 4 cm H_2_O, respectively. Values were adjusted whenever required. In cases of hypoxemia, with PaO_2 _≤ 60 mm Hg and/or SaO_2 _≤ 90%, EPAP was increased by 2 cm H_2_O until hypoxemia improved, as well as the IPAP level, to maintain the delta inspiratory pressure value. IPAP was increased or decreased according to the f/TV ratio, or in cases of hypoventilation with PaCO_2 _≥ 50 mm Hg, without a history of CO_2 _retention, IPAP was increased until hypoventilation improved with minimal air leakage. All patients received oxygen supplementation through a catheter connected to the nasal mask, with a flow of 5 L/min.

### Oxygen-mask group

Patients randomized to the oxygen-mask group received oxygen immediately after extubation through a facial mask with a flow of 5 L/min.

### Reintubation criteria

Reintubation required within a period of 48 hours after extubation was considered weaning failure in any one study group. The decision for reintubation was made by the staff physician at the ICU, in the persistent presence of one or more of the following criteria: systolic arterial pressure ≥ 180 mm Hg or ≤ 90 mm Hg, heart rate ≥ 140 beats/min, life-threatening arrhythmia, decreased level of consciousness or intense agitation requiring sedation, respiratory rate ≥ 30/min, PaO_2 _≤ 60 mm Hg or SaO_2 _≤ 90%, PaCO_2 _≥ 50 mm Hg, pH < 7.2, or significant difficulty in eliminating respiratory secretions.

### Follow-up

At baseline, demographic data (age and gender), Acute Physiology and Chronic Health Evaluation II (APACHE II) score, duration of mechanical ventilation before extubation, and diseases that led to acute respiratory failure were recorded for all patients. Heart rate, arterial blood pressure, respiratory rate, and hemoglobin saturation were monitored throughout the study for both groups. Three arterial blood gas analyses were carried out by radial artery puncture at 15 minutes, 2 hours, and 24 hours after extubation for all patients. Patients were followed up throughout their ICU and hospital stay. The need for reintubation was recorded, as well as the length of ICU stay and hospital mortality.

### Statistical analysis

*A priori *initial sample-size calculation (40 patients for each group) was made, aiming to show an absolute decrease in the rate of reintubation after 48 hours of 17.85% and a relative decrease in the rate of reintubation after 48 hours of 85% in the NIV group compared with the OM group. We considered the initial risk of 48 hours reintubation rate in the OM group of 21% (a usual finding in our ICU). This reflected our assumption that the reduction of the 48-hour reintubation ratio would decrease from 21% to 3.15%, as we informed the statistician that we expected a very high impact of noninvasive ventilation as an adjunctive weaning tool, according to our clinical experience with this ICU Brazilian population. The study power was 80%, and the α error was 5%. A first interim analysis was planned to be performed after a total of 40 patients to estimate the final sample size by an analyst blinded to the results and to the patients' outcomes. The Student *t *test was used for quantitative variables: age, days under mechanical ventilation before weaning, number of days in the ICU, and APACHE II. Repeated measures analysis of variance (ANOVA) with Bonferroni correction was used to compare consecutive measurements of pH, PaO_2_, PaCO_2_, SaO_2_, respiratory rate, heart rate, and mean arterial blood pressure. The Fisher Exact test was used to compare proportions. Kaplan-Meier curve was used to estimate hospital survival and the probability that the patients breathed without ventilator assistance, comparing the two study groups with the log-rank test. Relative risk and the number needed to treat (NNT) were also calculated. *P *< 0.05 was taken as the level of significance.

## Results

**Figure 1:**
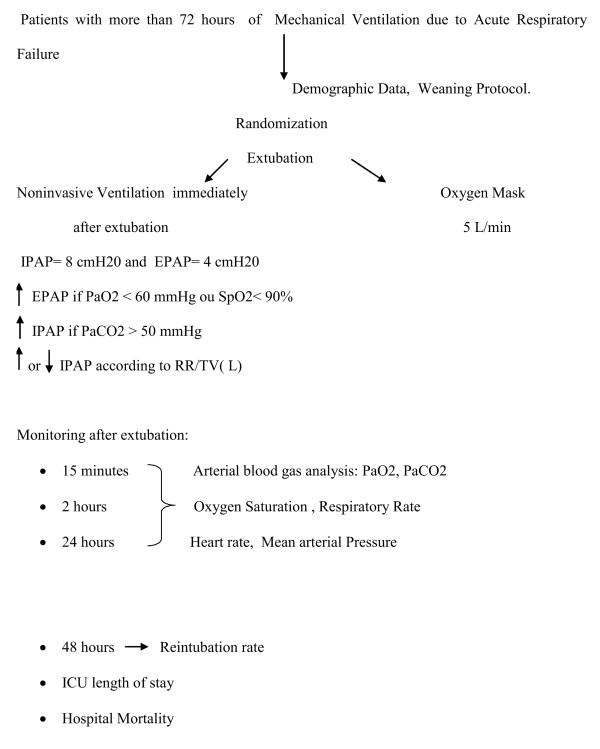
**Study design**.

**Figure 2:**
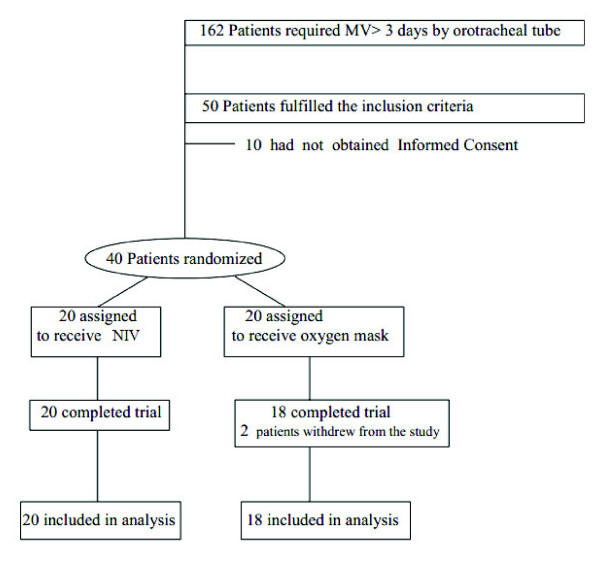
**Randomization of patients**.

Over the 12-month study period, a total of 162 patients admitted to the ICU required mechanical ventilation (orotracheal intubation) for a period greater than 3 days. Forty of them fulfilled the inclusion criteria and were randomized into the two groups. Twenty patients were randomized to the NIV group and underwent noninvasive ventilation by using a silicone nasal mask immediately after extubation; all 20 patients in this group finished the study, whereas 18 of the 20 patients who were randomized to the Oxygen Mask group and underwent nebulization with an oxygen mask after extubation finished the study (two patients withdrew from the study) (Figures 1 and 2).

Analysis of demographic data, days under invasive mechanical ventilation before weaning, APACHE II, and diseases that led to acute respiratory failure showed no statistically significant differences between groups (Table 1).

**Table 1:** Baseline characteristics and diseases that led to acute respiratory failure of the study groups

Variable	NIV (*n *= 20)	Oxygen mask (*n *= 18)	*P *value^a^
Gender (M/F)	14/6	12/6	1.0
Age (years), mean (SD)	50.79 (17.77)	48.88 (22.38)	0.77
Days of MV, mean (SD)	9.85 (8.05)	9.5 (6.06)	0.88
APACHE II, mean (SD)	16.90 (6.81)	15.28 (5.65)	0.43
Pneumonia, number (%)	16 (80)	16 (88.9)	0.66
COPD, number (%)	7 (35)	3 (16.7)	0.28
Abdominal surgery, number (%)	5 (25)	4 (22.2)	1.0
Sepsis, number (%)	4 (20)	2 (11.1)	0.66
Asthma, number (%)	2 (10)	1 (5.5)	1.0
Cardiac failure, number (%)	2 (10)	1 (5.5)	1.0

**^a^**Significant values with significance level of 0.05. APACHE II, Acute Physiology and Chronic Health Evaluation; COPD, chronic obstructive pulmonary disease; NIV, noninvasive positive-pressure ventilation.

**Figure 3:**
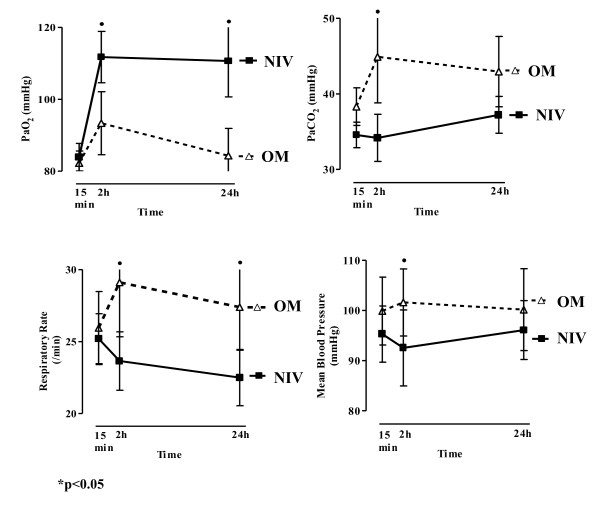
**PaO_2_, PaCO_2_, RR, and mean arterial pressure profile for the NIV and OM groups at the three different time points (15 minutes, 2 hours, and 24 hours)**.

Mean PaCO_2 _after 15 minutes from extubation was 34.56 ± 3.43 mm Hg in the NIV group and 38.31 ± 4.74 mm Hg in the OM group (*P *= ns). Mean PaO_2 _15 minutes after elective extubation was 83.2 ± 7.78 mm Hg in the NIV group and 82.23 ± 6.41 in the OM group (*P *= ns). Mean pH 15 minutes after extubation was 7.37 ± 0.03 in the NIV group and 7.38 ± 0.03 in the OM group. Mean respiratory rate was 25.2 ± 3.53/min in the NIV group and 25.95 ± 4.83/min in the OM group. Analysis of variance (ANOVA) showed a higher PaO_2_, lower PaCO_2_, respiratory rate, and mean blood pressure in the NIV group compared with the OM group during the 24-hour period (Figure 3).

**Figure 4:**
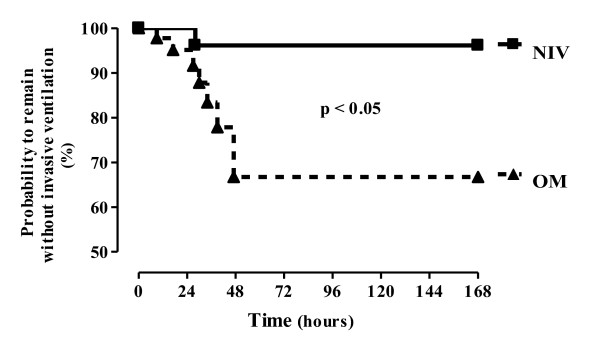
**Probability of avoiding reintubation after 168 study hours, by using the Kaplan-Meier curve, with a *P *value obtained with the log-rank test**.

The overall reintubation rate was 21%. Eight of the 38 evaluated patients were reintubated within 48 hours after extubation. The reintubation rate was different in each group. One patient of the 20 patients randomized to the NIV group was reintubated (5%), whereas seven of the 18 patients randomized to the OM group were reintubated (39%), *P *= 0.016 (Table 2 and Figure 4). This statistically significant difference was maintained even after the exclusion of patients with COPD (Table 2). The causes for reintubation in both groups are shown in Table 3. Relative risk for reintubation when using NIV after extubation was 0.13 (CI, 0.017 to 0.946), Absolute risk reduction (the difference in event rate between the control group and the study group) showed a decrease of 33.9%, and analysis of the number needed to treat was 3.

**Table 2:** Outcomes for the study groups

Outcomes	NIV (*n *= 20)	Oxygen mask (*n *= 18)	*P *value^a^
Reintubation, number (%)	1 (5%)	7 (39%)	0.016
Reintubation after excluding COPD, number (%)	0 (0) *n *= 13	5 (33%) *n *= 15	0.044
ICU length of stay, mean (SD)	16.8 (11.6)	18.4 (12.2)	0.681
Hospital mortality, number (%)	0 (0)	4 (22.2%)	0.041

*Significant values with significance level of 0.05. COPD, chronic obstructive pulmonary disease; ICU, intensive care unit; NIV, noninvasive positive-pressure ventilation.

**Table 3:** Causes of reintubation for the NIV and OM groups

	Respiratory muscle fatigue	Atelectasis	Bronchospasm	Depressed level of consciousness
90 mm Hg ≥ SAP ≥ 180 mm Hg	2 3		6	
HR ≥ 140 beats/min				
Severe arrhythmia				
RR ≥ 30 per minute	1 2 3	4 5	6	
PaO_2 _≤ 60 mm Hg or SaO_2 _≤ 90%	1 2 3	4 5	6	7 1^a^
PaCO_2 _≥ 50 mm Hg	1 2 3	4 5	6	7 1^a^
Difficulty in expectorating	1 2 3			7 1^a^

1, 2, 3, 4, 5, 6, 7, Seven patients in the Oxygen Mask group undergoing reintubation. ^a^Patient in the NIV group undergoing reintubation. HR, heart rate; NIV, noninvasive ventilation; RR, respiratory rate.

ICU length of stay was not statistically different between the groups, with a mean of 16.8 ± 11.6 days in the NIV group and 18.4 ± 12.2 days in the OM group (*P *= 0.681) (Table 2).

**Figure 5:**
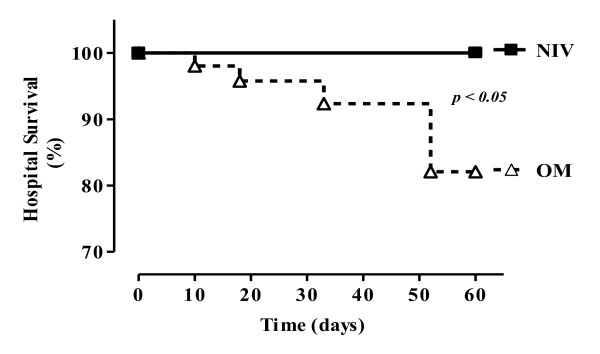
**Estimated hospital survival, by using the Kaplan-Meier curve**.

Hospital mortality rate showed a statistically significant difference between groups, with no deaths during hospitalization in the NIV group and four (22.2%) deaths in the OM group (*P *< 0.04; Table 2 and Figure 5). It is necessary to stress that all patients that died had been reintubated. The causes of death were pneumonia associated with sepsis in two patients, acute myocardial infarction in one patient, and multiple organ dysfunction syndrome in one patient.

Adverse effects related to the use of NIV were observed in two patients and were related to the nasal mask, consisting of ulceration at the nasal area with a good response to local treatment, and did not require discontinuation of NIV.

## Discussion

Recent relevant published evidence revealed that in patients with acute respiratory failure, early extubation with immediate application of NIV has a positive impact on important outcomes, turning to advantage when compared with continuous invasive weaning, especially in COPD patients that failed in spontaneous breathing trials [1-6]. Its use decreases the occurrence of ventilator-associated pneumonia, length of ICU and hospital stay, and total duration of mechanical ventilation, besides reducing patient mortality [1-6]. However, the benefit of using it in acute respiratory failure patients immediately after usual or planned extubation instead of the oxygen mask to prevent the occurrence of respiratory failure has not been totally established. One small trial assessed noninvasive positive-pressure ventilation after extubation and found no benefit [18]. This study has limited generalizability because it included a high proportion of patients with self-extubations. The present study, however, showed that patients undergoing invasive mechanical ventilation for more than 72 hours (our mean mechanical ventilation time was 9 days), owing to acute respiratory failure caused by different etiologies, had a lower reintubation rate if NIV was immediately applied after extubation when compared with those undergoing conventional therapy with an oxygen mask (5% versus 39%). Our hypothesis is that the major cause for the high success rate in the NIV group was the early application of the ventilatory technique, immediately after a programmed extubation, which probably kept the upper airways open, improving ventilation and oxygenation, thus preventing the overload of the respiratory muscles, the development of atelectasis, ventilation/perfusion disorders, and respiratory distress. A critical issue related to the success of NIV in our study was the adjustment of IPAP and EPAP levels according to each patient's needs. IPAP was adjusted for ventilation adequacy, whereas EPAP was adjusted for the maintenance of airways and alveolar stability. These individualized adjustments to the levels of NIV support may have had a pivotal role in the avoidance of reintubation in our population.

It is necessary to stress that NIV must be used immediately after extubation to avoid respiratory failure and consequent reintubation after elective extubation. Keenan and colleagues [19] showed that if NIV is administered to patients that develop respiratory distress after extubation to oxygen mask, NIV will not be able to prevent reintubation. Corroborating the results of Keenan and colleagues, Esteban and colleagues [20] showed that applying NIV to treat postextubation acute respiratory failure in nonselected populations may not be effective and could even be deleterious. NIV is not indicated now in cases that develop acute respiratory failure after extubation. In this situation, patients should be reintubated and mechanically ventilated.

Four recent RCTs suggested benefit from noninvasive positive-pressure ventilation after extubation in patients who were at high risk of deterioration [8,21-23]. High-risk patients were defined differently among the RCTs: (a) older than 65 years, cardiac failure as the cause of intubation, or APACHE II score greater than 12 at the time of extubation [21]; (b) more than one of the following: failure of consecutive weaning trials, chronic cardiac failure, arterial pressure of carbon dioxide greater than 45 mm Hg after extubation, more than one noncardiac comorbidity, or weak cough or stridor after extubation not requiring immediate intubation [22]; (c) acute exacerbation of COPD [23]; or (d) history of chronic respiratory disease with ventilation for more than 48 hours and hypercapnia during the spontaneous breathing trial [24]. Although the four trials defined higher risk differently, they reported consistent decreases in rates of reintubation (RR, 0.42; 95% CI, 0.25 to 0.70) and ICU mortality (RR, 0.35; 95% CI, 0.16 to 0.78), but less benefit in terms of hospital mortality (RR, 0.66; 95% CI, 0.42 to 1.04).

In our study, we aimed to analyze a mixed ICU population intubated and mechanically ventilated for more than 72 hours as the main risk factor. It is noteworthy that despite a lower 48-hour reintubation rate and mean blood pressure in the NIV group compared with the OM group along the 24-hour postextubation period (Figure 3), the NIV provided a better respiratory support to our patients in these critical moments after extubation.

In our study, relative risk analysis showed beneficial results of the use of NIV immediately after extubation. The use of NIV to avoid reintubation in patients with acute respiratory failure was approximately 8 times more frequent when compared with the oxygen mask. According to our results, the use of NIV after our weaning protocol avoided reintubation in one of every three patients when compared with the OM group, justifying its use immediately after extubation from invasive mechanical ventilation in patients with acute respiratory failure that needed more than 3 days of mechanical ventilation.

Another crucial issue related to our study results was the finding that the hospital mortality rate was higher in the OM group. It is noteworthy that all patients who died had been reintubated, and the causes of death were pneumonia associated with sepsis in two patients, acute myocardial infarction in one patient, and multiple organ dysfunction syndrome in one patient. This fact was previously reported in literature, indicating that reintubation is a risk factor for death in this population [24,25]. Therefore, all care should be taken to avoid reintubation after elective extubation in the ICU mechanical ventilation population setting.

With reference to the use of NIV as a preventive measure to avoid reintubation in patients on mechanical ventilation for more than 48 hours (in our study, 72 hours), Chien-Ling Su and colleagues [26] recently analyzed 406 randomized patients assigned to OM (204 patients) or NIV (202 patients). Reintubation rate at 72 hours was the primary outcome measure (in our study, 48 hours). The authors considered extubation failure if the patients still required the use of NIV at the end of 72 hours in the NIV group or any use of NIV within the first 72 hours after extubation in the OM group (in our study, the use of NIV in the oxygen group was not allowed after extubation and, in case of respiratory failure, our patients were reintubated). As Chien-Ling Su and colleagues used the NIV in the OM group to treat patients with acute respiratory failure after extubation, they could decrease the reintubation rate of their population. In our study, we used NIV during a 24-hour period after extubation, whereas, in the study of Chien-Ling Su and colleagues, they used the NIV only for 12 hours after extubation. In our study the control group failed mainly after 24 hours after extubation (Figure 4), meaning that we could avoid respiratory failure after 24 hours of extubation, whereas Chien-Ling Su could avoid reintubation by using early NIV ventilation after extubation in their control group. As the designs of the two studies were different, the results can be interpreted only by taking into account the different methods used.

Recently, Vaschetto and colleagues [27] reported a pilot study aimed to assess the feasibility of early extubation followed by immediate NIV compared with conventional weaning, in 20 randomized patients with resolving hypoxemic acute respiratory failure. They observed that it is possible to extubate hypoxemic acute respiratory failure patients early with no significant statistical differences in extubation failure, ICU and hospital mortality, tracheotomies, septic complications, days and rates of continuous sedation, and ICU length of stay. As most of our patients (80% in both groups) had pneumonia as the main cause of acute respiratory failure, this recent study supports our favorable results.

### Limitations

Certain limitations of this work must be recognized. We used nasal mask for the application of NIV after planned extubation. Whether the results with facial masks would be the same must be tested. Our studied population was small, but we finished the study after the first interim analysis because the primary objective of the study (prevent reintubation) was already achieved and was statistically significant (*P *= 0.016), and it was ethical to stop the study at that point. The problem of this early stop is that an overestimation of treatment effects could have occurred if our decision to stop the trial coincided with the random high in the treatment effect [28]. For that reason, further studies with a larger population must be carried out to confirm the favorable results obtained in this study and consequently, to expand the use of NIV to prevent reintubation in patients that need more than 3 days of invasive mechanical ventilation because of acute respiratory failure, provided that the NIV is applied immediately after extubation.

As our study was not blinded, a potential performance bias could have influenced our outcomes because the decision to reintubate the patients was left in the end to the discretion of the ICU attending physician, despite having reasonably objective criteria for reintubation. For this reason, we analyzed the reintubation rate after 168 hours or 7 days after extubation, and our results were maintained the same, making the potential performance bias more remote (Figure 4).

## Conclusions

Noninvasive ventilation compared with oxygen mask alone prevented reintubation and decreased hospital mortality if done immediately after planned extubation in our mixed ICU patients requiring invasive mechanical ventilation for more than 3 days because of acute respiratory failure.

## Key messages

• Noninvasive ventilation, if used immediately after planned extubation, reduced the reintubation rate in mixed ICU patients with respiratory failure in need of mechanical ventilation for more than 72 hours.

• Patients weaned by using noninvasive ventilation showed a higher PaO_2_, lower PaCO_2_, respiratory rate and mean blood pressure compared with those using the oxygen mask during the 24- hour period of observation.

• Patients weaned by using noninvasive ventilation had a significantly lower hospital mortality compared with patients weaned by using an oxygen mask.

## Abbreviations

ANOVA: analysis of variance; APACHE II: acute physiology and chronic health evaluation II; ARF: acute respiratory failure; BiPAP: bi-level positive airway pressure; CI: confidence interval; COPD: chronic obstructive pulmonary disease; EPAP: expiratory airway pressure; FIO_2_: oxygen inspiratory fraction; f/VT: ratio of respiratory rate and tidal volume in liters; ICU: intensive care unit; IPAP: inspiratory airway pressure; ISRCTN: International Standard Randomized Controlled Trial Number; NIV: noninvasive ventilation; NNT: number needed to treat; OM: oxygen mask; PaCO_2_: partial carbon dioxide arterial pressure; PaO_2_: partial oxygen arterial pressure; PaO_2_/FIO_2 _ratio: partial oxygen arterial pressure divided by oxygen inspiratory fraction; PEEP: positive end-expiratory pressure; pH: hydrogen ion concentration; PSV: pressure support ventilation; RCTs: randomized controlled trials; SaO_2_: arterial oxygen saturation; SIMV: synchronized intermittent mandatory ventilation; S/T-D: spontaneous timed mode.

## Competing interests

The authors declare that they have no competing interests.

## Authors' contributions

SRPO, SMAL, and CSVB designed the randomized clinical protocol, analyzed the data, and drafted and reviewed the final version of the paper. SRPO, SMAL, HSS, MD, LTT, and AMAV collected the data. GPPS, MBPA, and CRRC participated in the discussion of results, drafting and reviewing of the paper. All authors read and approved the manuscript for publication.

## Authors' information

This study was the PhD thesis of SRPO presented at the University of São Paulo Medical School, and CSVB was her orientator. SRPO, SMAL (Head of the Unit) and HSS are staff physicians at the ICU of Hospital de Base São José do Rio Preto, State of São Paulo, Brazil. MD, LTT, and AMAV are staff respiratory physiotherapists at the ICU of Hospital de Base São José do Rio Preto, State of São Paulo, Brazil. CSVB, MBPA, GPPS, CRRC (Head of the Unit) are staff physicians at the Respiratory ICU of Hospital das Clinicas of the University of São Paulo, Brazil.
